# 

**Published:** 1905-11

**Authors:** 


### BOOKS RECEIVED.

A Textbook of Practical Theraputics, with Especial Reference to the Application of Remedial Measures to Disease and theirEmployment upon a Rational Basis. By Hobart Amory Hare, M.D., B.Sc., Professor of Theraputics and Materia Medica in the Jefferson Medical College, Philadelphia. With special chapters by G. E. de Schweinitz, Edward Martin ,and Barton C. Hirst. Eleventh edition, enlarged, thoroughly revised, and largely rewritten. Octavo, 910 pages, with 113 engravings and 4 full-page colored plates. Philadelphia and New York: Lea Brothers and Company. 1904. (Price: cloth, $4.00; leather, $5.00; half morocco, $5.50 net prices.)

A Manual of Diseases of Infants and Children. By John Ruhräh, M.D., Clinical Professor of Diseases of Children, College of Physicians and Surgeons, Baltimore. 12mo volume of 404 pages, fully illustrated. Philadelphia and London: W. B. Saunders & Co., 1905. Flexible leather, $2.00 net.

Dissecting Manual, based on Cunningham's Anatomy. By W. H. Rockwell, Jr., M.D., Formerly Assistant Demonstrator of Anatomy in the College of Physicians and Surgeons, Columbia University, New York. Small octavo, pp. 306. New York: Williaw Wood & Co. 1905. (Price: $2.00)

A Text-Book of Physiology: for Medical Students and Physicians. By William H. Howell, Ph..D., M.D., LL.D., Professor of Physiology, Johns Hopkins University, Baltimore. Octavo volume of 905 pages, fully illustrated. Philadelphia and London: W. B. Saunders & Co., 1905. Cloth, $4.00 net; Half Morocco, $5.00 net.

The Principles of Bacteriology. A Practical Manual for Students and Physicians. By A. C. Abbott, M.D., Professor of Hygiene, University of Pennsylvania. Seventh edition, enlarged and thoroughly revised. In one 12mo. volume of 690 pages, with 100 illustrations, of which 24 are colored. Lea Brothers & Co., New York and Philadelphia. 1905. (Cloth, $2.75 net.)

THE publication of this Journal has again been delayed as a result of the printers' "strike." We promise in future more promptitude.

A. T. BROWN PRINTING HOUSE

